# *Mycoplasma pneumoniae* Infections and Primary Immune Deficiencies

**DOI:** 10.1155/2022/6343818

**Published:** 2022-07-08

**Authors:** Dimitri Poddighe, Erkan Demirkaya, Vitaliy Sazonov, Micol Romano

**Affiliations:** ^1^Department of Medicine, Nazarbayev University School of Medicine (Nur-Sultan), Nur-Sultan, Kazakhstan; ^2^Clinical Academic Department of Pediatrics, National Research Center of Mother and Child Health, University Medical Center, Nur-Sultan, Kazakhstan; ^3^Division of Pediatric Rheumatology, Schulich School of Medicine & Dentistry, University of Western Ontario, London, Canada; ^4^Department of Epidemiology and Biostatistics, Schulich School of Medicine & Dentistry, University of Western Ontario, London, Canada; ^5^Department of Biomedical Sciences, Nazarbayev University School of Medicine (Nur-Sultan), Nur-Sultan, Kazakhstan

## Abstract

*Mycoplasma pneumoniae* (*M*. *pneumoniae*) is one of the leading causes of community-acquired pneumonia in children and is also implicated in a variety of reactive extrapulmonary diseases. Recurrent and/or severe respiratory infections are one of the most frequent manifestations of several types of primary immunodeficiency. Here, we reviewed the medical literature to assess the potential relevance of *M*. *pneumoniae* in the infections observed in children affected with combined, humoral, and innate primary immune deficiencies. *M*. *pneumoniae* does not result to be epidemiologically prevalent as a cause of pneumonia in children affected by primary immunodeficiencies, but this infection can have a persistent or severe course in this category of patients. Indeed, the active search of *M*. *pneumoniae* could be useful and appropriate especially in children with humoral immune deficiencies. Indeed, most cases of *M*. *pneumoniae* infection in primary immunodeficiencies are described in patients affected by a/hypo-gammaglobulinemia.

## 1. Introduction

*Mycoplasma pneumoniae* (*M*. *pneumoniae*) causes upper and lower respiratory tract infections [1]. Even though upper respiratory infections caused by *M*. *pneumoniae* can be mild and have unspecific symptoms (and thus frequently overlooked), this microorganism is one of the leading causes of community-acquired pneumonia (CAP), especially in children. Indeed, up to 10% of *M*. *pneumoniae*-infected children develop pneumonia, and *M*. *pneumoniae* can account for up to 20% of CAP cases in school-aged children [2].

In the landscape of pediatric respiratory disorders, *M*. *pneumoniae* has been also recognized as a triggering factor for wheezing or asthma exacerbations [2, 3]. Moreover, *M*. *pneumoniae* infections have been also implicated in several extra-respiratory diseases (*M*. *pneumoniae* extrapulmonary diseases, MpEPDs), which may affect a variety of organs, including skin, mucosae, muscles, joints, heart, and central nervous system. Even though the pathogenesis of MpEPDs has not been fully elucidated yet (and is likely to be variable according to different types of extrapulmonary manifestations), it is supposed to be immune-mediated [4–6]. Recent studies suggested that MpEPDs, as well as pulmonary complications, may be promoted by delayed antibiotic treatment and/or microbial persistence due to antibiotic-resistant strains, [7–10] whereas other authors evidenced some correlation with immunological aspects, such as increased levels of serum IgE and/or the presence of a Th2-polarized immunological environment [10–14].

Therefore, *M*. *pneumoniae* seems to have a complex (and not fully elucidated) interaction with the host's immune system. Although *M*. *pneumoniae* is one of the leading causes of CAP [1, 2], its relevance in the setting of primary immunodeficiencies (PIDs) seems to be poorly investigated so far. Indeed, PIDs are rare disorders determined by hundreds of specific genetic defects, which are associated with variable susceptibility to different types of microbial agents [15]. In general, respiratory infections and, in detail, pneumonia are among the most frequent infectious manifestations of immunocompromised children [16].

Here, we reviewed the medical literature to assess the potential relevance of *M*. *pneumoniae* in the infections observed in children with PIDs and, thus, provide a background of knowledge to plan further and specific clinical investigations.

## 2. Combined Primary Immunodeficiencies

Schematically, this category includes severe combined immunodeficiencies (SCID) and combined immunodeficiencies with associated or syndromic features [15].

SCID patients usually present within the first months of life and develop severe and recurrent infections caused by opportunistic pathogens, in addition to other manifestations, such as skin rashes, chronic diarrhea, and failure to thrive. Therefore, these infections often cause a fatal outcome in SCID patients, unless hematopoietic stem cell transplantation (HSCT) can be timely performed. Therefore, there are no reports of *M*. *pneumoniae* infections in these patients [16, 17]. Other combined immunodeficiencies (e.g., DNA repair defects, immunodeficiencies with congenital thrombocytopenia, thymic defects, immuno-osseous dysplasia, hyper-IgE syndromes, etc.) are less severe and, indeed, have a later onset in childhood with recurrent infections, which can be opportunistic or not [16].

*M*. *pneumoniae* infections are not reported as a significant cause of morbidity in most of these combined immunodeficiencies [18]. However, there are some reports in patients affected with hyper-IgE syndromes. Chandesris et al. described the molecular and clinical features of 60 patients with heterozygous STAT3 mutations, then affected with autosomal dominant hyper-IgE syndrome because of STAT3 deficiency (Job's syndrome). These patients are generally characterized by severe bacterial infections leading to severe pneumonia, pulmonary abscesses, and pneumatoceles. In this case series, 3 patients with *M*. *pneumoniae* infection were reported: in detail, *M*. *pneumoniae* was associated with other germs (*S*. *aureus* and *S*. *pneumoniae*) in 2 cases, but *M*. *pneumoniae* was the only etiologic agent identified in one case who developed severe pneumonia with pleural effusion, bronchiectasis, and pneumatocyst [19].

Hantz et al. reported another case of severe and persistent pneumonia due to *M*. *pneumoniae* in the context of Job's syndrome. This 11-year-old boy developed interstitial pneumonia needing respiratory support, and *M*. *pneumoniae* was the only bacterial agent that could be detected by PCR in the sputum and nasopharyngeal swab; notably, this patient resulted negative for all 15 tested viral pathogens (including influenza virus A/B, parainfluenza viruses, respiratory syncytial virus, coronavirus 229E/NL63/OC43, bocavirus, metapneumovirus, rhinoviruses, enterovirus, and adenovirus), except for parainfluenza type 3 virus, which anyway was positive only at the disease onset and was not detected anymore during following 4-week clinical course. Moreover, in this clinical case, the authors showed the occurrence of macrolide resistance in the same *M*. *pneumoniae* isolate detected during macrolide treatment [20].

Indirect evidence of the impact of combined immunodeficiency on *M*. *pneumoniae* susceptibility may derive from the analysis of cases in children infected with HIV. Nadagir et al. observed a 32% prevalence of *M*. *pneumoniae* infections among HIV seropositive children with respiratory manifestations [21]. Some authors suggested that CD4^+^ T cell depletion associated with advanced HIV disease may enhance *M*. *pneumoniae*-related lung disease [22, 23]. A case of recurrent *M*. *pneumoniae* respiratory infections in an HIV-positive child was described by Watson et al., who suggested including *M*. *pneumoniae* in the diagnostic work-up of HIV-positive children developing pneumonia or recurrent/persistent respiratory manifestations [24]. Indeed, Merida-Vieyra et al. recently described a cohort of 154 children with CAP treated in a tertiary care hospital. They detected *M*. *pneumoniae* in 26.6% of their patients and, notably, 83% of them had one or more underlying chronic disease, including 7 patients affected with immunodeficiency. Among them, there were three patients with AIDS and one of them died; however, this patient had an additional comorbidity, namely, a congenital heart disease. Unfortunately, most studies on CAP in children excluded patients with underlying immune defects and/or infected with HIV, which prevents us from any conclusive comments [25].

## 3. Predominantly Antibody Primary Immunodeficiencies

This category of PIDs mainly relies on B-cell defects and represents the most consistent part of PID diagnoses (at least 50%). In general, PIDs characterized by a prevalent humoral defect show increased susceptibility to respiratory tract infections. These patients usually start manifesting this problem around or after 6 months of age: recurrent and/or severe episodes of otitis media, sinusitis, and pneumonia are often observed. Basically, these PIDs are characterized by reduced or absent serum immunoglobulin levels. Notably, patients with humoral immunodeficiency can be also diagnosed in adulthood [16, 26].

The most frequent form of PID in this category is represented by IgA deficiency, which is also the most common PID [27]. The most severe forms of humoral PIDs are those characterized by absent (or profoundly decreased) B lymphocytes (as it happens in X-linked agammaglobulinemia, XLA) or by an important reduction in at least 2 serum immunoglobulin isotypes, despite normal or low number of B cells (like in common variable immunodeficiency, CVID). A specific category is represented by hyper-IgM syndromes, where a severe reduction in serum IgG and IgA is actually associated with normal or elevated IgM and normal numbers of B cells [15, 16].

As already mentioned, recurrent and/or severe and/or deep respiratory infections (including pneumonia) are usually the clinical hallmark of PIDs with prevalent humoral defects; as regards bacterial infections, *Streptococcus pneumoniae* and *Haemophilus influenzae* are the most frequent causal agents [16]. However, *M*. *pneumoniae* may be also implicated in the respiratory infections observed in these immune-depressed patients. Indeed, the first observations in this regard date back to 1973, when Foy et al. described 4 pediatric patients (one diagnosed with XLA and three with CVID) who developed severe pneumonia and/or prolonged respiratory illness due to *M*. *pneumonia* infections [28]. Eventually, Taylor-Robinson et al. first reported in 1980 that a 7-year-old boy with hypogammaglobulinemia developed severe pneumonia caused by *M*. *pneumoniae* infection, which had a prolonged clinical course despite several different antibiotic treatments [29]. Actually, the same group previously also described the repeated isolation of *M*. *pneumoniae* from the knee joint of an adult patient with chronic polyarthritis who was also affected by an unspecified form of hypogammaglobulinemia [30]. Other reports described extrapulmonary infections (septic arthritis, osteomyelitis, encephalitis, and skin abscess) by *M*. *pneumoniae* in adult patients affected with moderate-severe hypogammaglobulinemia [31–34]. Moreover, unusual and/or prolonged infections by other *Mycoplasma* spp. have been reported in patients with hypogammaglobulinemia [35–37]. Roifman et al. reported that, during a 3-year follow-up period, they diagnosed mycoplasmas infections (including *M*. *pneumoniae* in 2 patients) in 13 of their 23 patients affected with hypogammaglobulinemia. Notably, both patients with pneumonia caused by *M*. *pneumoniae* were children [38]. Conversely, Kainulainen et al. found no patients with *M*. *pneumoniae* infection in their case series of 14 patients affected with primary hypogammaglobulinemia who underwent bronchoalveolar lavage fluids (including 3 pediatric patients; XLA: *n* = 3; CVID, *n* = 2) [39].

Recent studies investigated the importance of humoral immunity in the host's defense against *M*. *pneumoniae* in both mice and children, which suggested the role of *M*. *pneumoniae*-specific antibodies in clearing this infection from the lungs. In detail, the IgG response to *M*. *pneumoniae*-derived proteins (rather than *M*. *pneumoniae* glycolipid-specific IgM and IgG antibodies) seems to be important for the pulmonary clearance of this microorganism [40–42].

As regards other B-cell immune defects, Cabral-Marques et al. described the infectious profile in 58 hyper-IgM patients included in the Registry of the Latin American Society for Immunodeficiencies (LASID). Among them, they reported one patient affected with CD40L deficiency who developed pneumonia by *M*. *pneumoniae* [43].

Finally, the aforementioned study by Merida-Vieyra et al. (describing a cohort of children with CAP) included 7 patients affected with immunodeficiency, as already mentioned. In detail, in addition to three patients with AIDS, three PID cases were represented by antibody defects (including two cases of hypogammaglobulinemia and one case of agammaglobulinemia); indeed, the fourth case of PID was affected by hyper-IgE syndrome [25].

## 4. Innate Immunity Defects

Congenital disorders of phagocyte number and function and complement deficiencies are the main PIDs included in this category [15]. Children affected with these disorders often develop unusual infections which are difficult to be eradicated. Children affected with phagocyte disorders (including chronic granulomatous disease, CGD) are prone to have pyogenic bacterial and fungal infections of the respiratory tract, but also at the level of the skin and internal organs. Patients with complement deficiencies can show severe or recurrent infections caused by encapsulated bacteria, but they also develop systemic autoimmune manifestations which may resemble lupus erythematosus, probably related to the efferocytosis impairment [16, 44].

Overall, there is no significant evidence that *M*. *pneumoniae* may be implicated in severe or recurrent pulmonary disease in patients affected with innate immunity defects. For instance, as regards CGD specifically, Song et al. and Kato et al. reviewed the infectious burden in these children: they retrieved only one dated report describing a case of pneumonia due to *M*. *pneumoniae* [45, 46].

More recently, Salvator et al. retrospectively assessed the pulmonary manifestations in the French national cohort of adult patients with CGD: they could identify the causative pathogen of bacterial pneumonia in 19 cases (63% of their cohort) and the etiology of the pulmonary disease was linked to *M*. *pneumonia* in one case only [47].

There are no specific case reports or clinical investigations analyzing *M*. *pneumoniae* infections in children affected with congenital neutropenia [24, 48, 49]. Notably, there is no evidence that acquired neutropenia in children with cancer significantly predisposes to *M*. *pneumoniae* pneumonia; conversely, recent studies showed a correlation between neutrophil number and extravasation into the lungs with the severity of *M*. *pneumoniae* pneumonia in this clinical setting [50, 51]. Indeed, Banov et al. reported only 3 cases of *M*. *pneumoniae* infection in 30 patients developing neutropenia-associated pneumonia in a cohort of 463 children who were treated for malignancy or received a hematopoietic stem cell transplant for a non-neoplastic disease [52].

Similarly, no severe cases of pneumonia by *M*. *pneumoniae* were reported in patients with primary complement deficiency. However, *M*. *pneumoniae* can trigger severe complications in children having a constitutional dysregulation of the alternative complement pathway; indeed, *M*. *pneumoniae* is reported as an unusual cause of atypical hemolytic uremic syndrome (aHUS) [53–55]. Mutations in complement factor H (CFH), complement factor I (CFI), complement factor B (CFB), MCP/CD46, and C3 account for about 50% of known genetic alterations in patients with aHUS [56].

## 5. Conclusion

*M.pneumoniae* does not result to be an epidemiologically prevalent cause of pneumonia in children affected with PIDs, but this infection can have a persistent or severe course in this category of patients. Indeed, the description of severe and persistent *M*. *pneumoniae* infection is mainly limited to case reports and small case series; there are very few clinical studies focused on *M*. *pneumoniae* infections (including both pneumonia and extrapulmonary manifestations) in children affected with PIDs. Therefore, further studies are required to precisely assess the real burden of this *M. pneumoniae* in these different PID-related pathological settings. The active search of *M*. *pneumoniae* could be useful and appropriate especially in children affected with humoral PIDs who develop pneumonia or persistent respiratory diseases: indeed, most cases of *M*. *pneumoniae* infection in PIDs are described in patients with a/hypo-gammaglobulinemia.

## Data Availability

Not applicable.
